# Caspase-1-Independent Interleukin-1β Is Required for Clearance of *Bordetella pertussis* Infections and Whole-Cell Vaccine-Mediated Immunity

**DOI:** 10.1371/journal.pone.0107188

**Published:** 2014-09-08

**Authors:** David E. Place, Sarah J. Muse, Girish S. Kirimanjeswara, Eric T. Harvill

**Affiliations:** 1 Department of Veterinary and Biomedical Sciences, Pennsylvania State University, University Park, Pennsylvania, United States of America; 2 Graduate Program in Immunology and Infectious Diseases, Pennsylvania State University, University Park, Pennsylvania, United States of America; 3 Graduate Program in Biochemistry, Microbiology and Molecular Biology, Pennsylvania State University, University Park, Pennsylvania, United States of America; University of Iowa Carver College of Medicine, United States of America

## Abstract

Whooping cough remains a significant disease worldwide and its re-emergence in highly vaccinated populations has been attributed to a combination of imperfect vaccines and evolution of the pathogen. The focus of this study was to examine the role of IL-1α/β and the inflammasome in generation of the interleukin-1 (IL-1) response, which is required for the clearance of *Bordetella pertussis*. We show that IL-1β but not IL-1α is required for mediating the clearance of *B. pertussis* from the lungs of mice. We further found that IL-1β and IL-1R deficient mice, compared to wild-type, have similar but more persistent levels of inflammation, characterized by immune cell infiltration, with significantly increased IFNγ and a normal IL-17A response during *B. pertussis* infection. Contrary to expectations, the cleavage of precursor IL-1β to its mature form did not require caspase-1 during primary infections within the lung despite being required by bone marrow-derived macrophages exposed to live bacteria. We also found that the caspase-1 inflammasome was not required for protective immunity against a *B. pertussis* challenge following vaccination with heat-killed whole cell *B. pertussis,* despite IL-1R signaling being required. These findings demonstrate that caspase-1-independent host factors are involved in the processing of protective IL-1β responses that are critical for bacterial clearance and vaccine-mediated immunity.

## Introduction

One of the critical initial mediators of inflammation is interleukin-1 (IL-1), which has pleiotropic effects in the body. Initially discovered as an immune component involved in induction of fever responses, the role of this cytokine has expanded to include chemokine induction, vascular permeability, expression of adhesion molecules, T cell differentiation, and has further been implicated in a number of pathologic conditions [1]–[6]. The requirement for IL-1 during infections has been studied in multiple infection models including *Shigella flexneri, Salmonella enterica, Staphylococcus aureus, Candida albicans,* influenza and *Bordetella pertussis* [7]–[12]. IL-1 mediated protection against extracellular pathogens often occurs through the production of IL-6, which polarizes CD4^+^ T cells to become IL-17-secreting Th17 cells, which can recruit neutrophils to the site of infection and mediate bacterial killing [13], [14]. For example, *B. pertussis* infection leads to the recruitment of specific Th17 cells that were recently shown to be dependent upon IL-1R signaling [10], [15], [16].

The most well characterized members of the IL-1 family include IL-1α, IL-1β, IL-18, and IL-33. The latter three cytokines are processed into their active form by the caspase-1 containing inflammasome complex [1], [17]. The IL-1α and IL-1β molecules both bind the IL-1R and exert similar downstream activities; however, their tissue localization and methods of activation differ, suggesting they may have distinct roles in immunity [1].

The induction of IL-1 is tightly regulated and requires two activation signals to produce the active IL-1α and IL-1β. During infection, expression of IL-1β is often induced downstream of the Toll-like receptors through translocation of the transcription factor NF-κB [18]. Following expression of the precursor proteins in the cytosol, a second signal is often required to cleave the proteins into their active mature forms [19], [20]. In the case of IL-1α, a calcium-activated cysteine protease, calpain, cleaves the precursor into its active form. Additionally, the IL-1α precursor has been shown to translocate to the nucleus to regulate expression of IL-6, IL-8, TNFα and IFNγ or can be released from necrotic cells as a danger signal [21]–[24]. The processing of IL-1β is regulated by the inflammasome, a complex of proteins including an adapter protein, ASC, and Nod-like receptors (NLRs) that, upon stimulation, assembles and activates pro-caspase-1 [17], [25]. Following activation, the cysteine protease caspase-1 cleaves the 31 kDa pro-IL-1β into its active 17 kDa form [26]. Recently, NLRP3 was shown to be a key component of the inflammasome, which is activated by the pore-forming *Bordetella pertussis* adenylate cyclase toxin through K^+^ efflux. Because the IL-1R signaling pathway is required for clearance of *B. pertussis* and rapid vaccine-mediated immunity, it was suggested that NLRP3-inflammasome activation of IL-1β would be required for controlling infection [10], [27].

Given that *B. pertussis* infections are highly transmissible, affecting 50 million people worldwide every year and re-emerging in many countries as a result of adaptation, and the limitations of the current generation of vaccines, it is critical to understand how the immune system processes IL-1 during infection by this particular respiratory pathogen [28]–[30]. In this study we focus on the factors upstream of the IL-1R during *B. pertussis* infection. Here we have found that IL-1β was required for clearance of *B. pertussis* and could be processed into its active form in a caspase-1 independent manner in the murine lung. Our vaccination results also show that IL-1 related signaling is important for protective immunity to *B. pertussis* and can be initiated independently from caspase-1, which has been shown to be activated by the current alum adjuvant in acellular pertussis vaccines. Future studies looking at alternative cleavage mechanisms will help to identify how IL-1β is processed during infection. Increasing our understanding of the regulation and function of IL-1 signaling against respiratory pathogens has broad implications for control of disease and improvement in vaccine design.

## Materials and Methods

### Mouse Strains

C57BL/6J, IL-1R^−/−^ (B6.129S7-*Il1r1^tm1Imx^*/J), and IL-18^−/−^ (B6.129P2-*Il18^tm1Aki^*/J) mice were purchased from The Jackson Laboratories (Bar Harbor, ME). Caspase-1^−/−^11^−/−^ mice were obtained from Dr. Richard A. Flavell (Yale School of Medicine). IL-1α (B6.129-*Il1a^tm1Yiw^*) and IL-1β-deficient mice (B6.129-*Il1b^tm1Yiw^)* on a C57BL/6J background were provided by Dr. Lloyd S. Miller (University of California, Los Angeles) and originally produced by Dr. Yoichiro Iwakura [31]. All mice were maintained in specific pathogen-free conditions at the Pennsylvania State University animal care facilities.

### Bacterial Strains and Cell Lines

*Bordetella pertussis* infections were performed using the streptomycin-resistant derivative of Tohama I strain 536 [32]. Bacteria were grown on Bordet-Gengou (BG) agar (HiMedia) supplemented with 15% defibrinated sheep blood (Hema Resources & Supply, Inc.) and 20 µg/mL streptomycin (Sigma-Aldrich) or shaking overnight in Stainer-Scholte broth supplemented with 500 µg/mL heptakis (Sigma-Aldrich) at 37°C [33].

Bone marrow-derived macrophages (BMDMs) were generated from bone marrow isolated from the femur and tibia as previously described, with minor modifications [34], [35]. Briefly, bones were crushed in a 70 µm nylon cell strainer (BD Biosciences), pelleted and resuspended before seeding plates. Cells were grown on bacterial grade petri dishes in 10 mL DMEM containing 2 mM GlutaMAX, 1 mM HEPES (Life Technologies), 10% FCS (Gibco, HyClone) and 20% M-CSF containing L929 cell supernatant. An additional 5 mL of medium was added at day 3, and cells were collected on day 6 with 1 mM PBS-EDTA and seeded into 24-well tissue culture-treated plates overnight.

### Mouse Infection

Mice were lightly anesthetized with isoflurane (Abbott Laboratories) and bacteria (5×10^5^ CFU) were delivered into the respiratory tract in a 50 µL droplet of sterile phosphate-buffered saline (PBS) via the external nares. Mice were euthanized by carbon dioxide inhalation and dissected in a biosafety cabinet with aseptic technique. Serial dilutions of the inoculum were plated on BG-blood agar containing 20 µg/mL streptomycin incubated for 4 days at 37°C to confirm the infection dose and lungs were homogenized in ice-cold sterile PBS at 30 minutes post-inoculation for day 0. Similarly, at indicated time points post-inoculation, lungs were excised and homogenized in 1 mL of sterile PBS. Aliquots were taken from this homogenate and either centrifuged and stored at −20°C for ELISAs and immunoblots or serially diluted ten-fold in sterile PBS with 100 µL plated for CFU counts to determine bacterial colonization with a limit of detection of 10 bacteria per lung.

### Flow Cytometry

Mouse lungs were collected from euthanized mice in 5 mL DMEM with 10% FCS and passed through a 70 µm cell strainer using a 3 mL syringe plunger. Cells were pelleted and resuspended in 2 mL 0.84% ammonium chloride to lyse red blood cells. Cells were blocked with anti-CD16/32 (93) and stained with fluorophore-conjugated CD45 (30-F11), CD3 (17A2), CD4 (GK1.5), CD8a (53–6.7), CD11b (M1/70), Ly6G (1A8), and F4/80 (BM8) from BioLegend and BD Biosciences, fixed in 2% paraformaldehyde, and analyzed on a BD LSR II flow cytometer. All analyzed cell populations were gated on FSC-A/FSC-H to exclude doublets and on CD45^+^ cells. Analysis was performed with FlowJo (Tree Star, Inc.).

### Histology

Mouse lungs were inflated with approximately 2 mL formalin intratracheally, collected in 5 mL formalin, embedded in paraffin wax and sectioned in 5 µm slices, and stained with hematoxylin and eosin (H&E) at the Huck Microscopy & Histology Core Facilities at The Pennsylvania State University. Two blinded, independent scorings were performed on histology sections based on previously established criteria including peribronchiolar inflammation, with percent bronchioles and degree of inflammation scored separately, perivascular inflammation, and intra-alveolar inflammation [36]. Images were collected with an Olympus BX51 scope with ProGres software.

### Cell culture infections

Bone marrow-derived macrophages were seeded in 24-well or 6-well tissue culture treated plates (Costar) and allowed to adhere overnight at 37°C in 5% CO_2_. Assay solutions were freshly prepared. Cells were washed and indicated wells were primed with 100 ng/mL *E. coli* 011:B4 LPS (Sigma-Aldrich) for three hours. After three hours, all wells were aspirated and replenished with fresh media, media containing 5 mM ATP (MPBiomedicals), or media containing live *B. pertussis* diluted from overnight cultures. Prior to collection of supernatant, plates were centrifuged at 500×*g* for 5 minutes.

### ELISAs and Immunoblots

Mouse lungs were collected in ice-cold PBS, homogenized, centrifuged, and supernatant was removed and frozen at −20°C until analysis. Cell culture supernatants were collected by spinning down plates at 500×*g* for 5 minutes and carefully pipetted into fresh plates or cell lysates were lysed in RIPA buffer (50 mM Tris-HCl pH 8, 150 mM NaCl, 1% IGEPAL, 0.5% sodium deoxycholate) and stored at −20°C. For measuring IL-1β and IL-10, OptEIA kits (BD Biosciences) were used according to manufacturer recommendations. IL-17 and IFNγ were measured by paired capture/detection antibodies (R&D Systems) or the DuoSet ELISA Development System (R&D Systems). Standard curves were generated with a 4-parameter logistic curve-fit and optical density (450 nm–570 nm) was interpolated to determine concentration using Gen 5 software and a Biotek Epoch Plate Reader.

For immunoblotting, lung homogenates or cell culture lysates were diluted in 2X Laemmli sample buffer (BioRad). Cell culture supernatants were concentrated by a methanol/chloroform precipitation method described previously [10]. Samples were boiled 5 minutes, run on a 12% TGX pre-cast gel and transferred to a polyvinylidene difluoride (PVDF) membrane with the Trans-Blot Turbo mixed molecular weight program (BioRad) and blocked in 5% bovine serum albumin (Sigma-Aldrich) prior to detection with Abcam anti-IL-1β (ab9722) and secondary goat anti-rabbit-HRP (Jackson Immunoresearch) utilizing the Immobilon chemiluminescence detection reagent (Millipore). Images were taken with the ChemiDoc XRS+ System (BioRad).

### Vaccination

Heat-killed bacteria vaccine was generated from overnight cultures of *B. pertussis* strain 536 following growth in Stainer-Scholte medium. Bacteria were washed in PBS and diluted to 10^9^ bacteria/mL and incubated at 65°C for 30 minutes to heat-kill bacteria which was confirmed by plating on BG-blood agar. Vaccine was stored at −20°C before use. Vaccination was performed by intraperitoneal injection of 200 µL 28 and 14 days prior to the challenge infection (5×10^5^ CFU).

### Statistical Analyses

Significant differences between groups were determined using Student’s t-test, one-way ANOVA with Dunnet’s or Tukey’s post-hoc test or the nonparametric Kruskal Wallis test with Dunn’s post-hoc test for significance using GraphPad Prism with *p* values <0.05 considered significant.

### Ethics Statement

All animal experiments were carried out by following recommendations and approval from the Pennsylvania State University Animal Care and Use Committee (protocols 39724 & 40029) with great care taken to minimize suffering of animals.

## Results

### IL-1β is required for efficient clearance of *B. pertussis*

Respiratory tract infection by *B. pertussis* induces the production of critical inflammatory mediators including IL-1[37]. The importance of IL-1 during *B. pertussis* infections to date has been ascribed to promoting development of IL-17 producing CD4^+^ T helper (Th17) cells and the recruitment and activation of neutrophils and macrophages [27], [38], [39].

**Figure 1:**
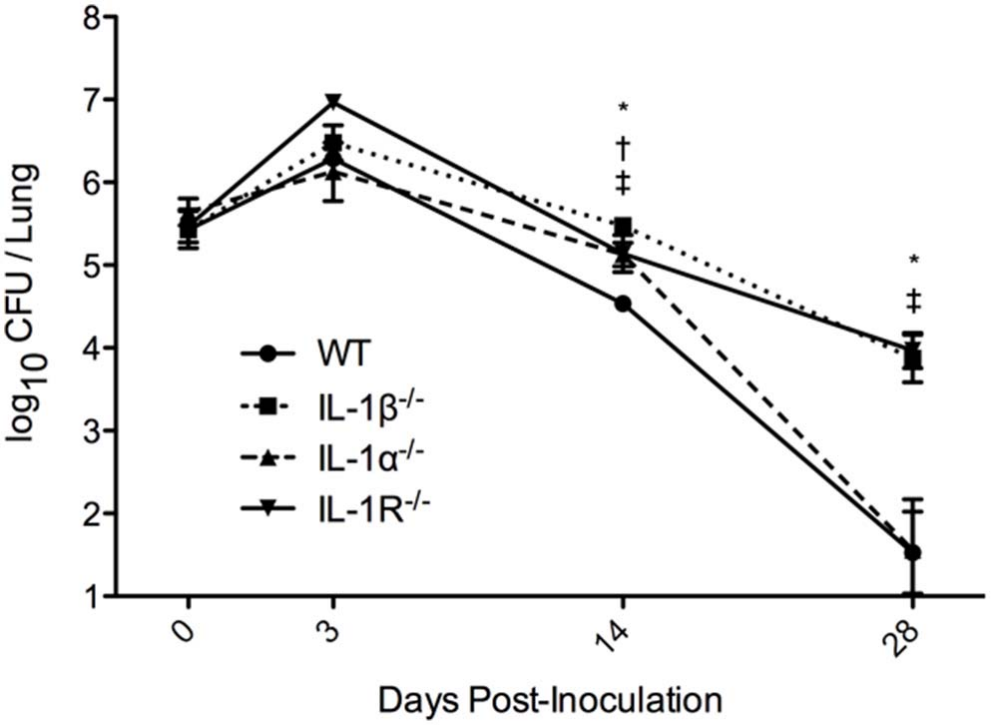
Bacterial clearance is mediated by IL-1β and IL-1R but not IL-1α. Groups of 5–8 mice were inoculated with *B. pertussis* (5×10^5^ CFU) suspended in a 50 µL droplet of PBS and euthanized at indicated time points to determine bacterial CFUs in lung homogenates. The mean ± standard error was graphed for each group at the specified time-point and is representative of three independent experiments. Significance was calculated using one-way ANOVA with Dunnet’s multiple comparison post-hoc analysis. *p<0.05, **p<0.01; WT vs IL-1β^−/−^ (*), vs IL-1α^−/−^ (†), vs IL-1R^−/−^ (‡).

While both IL-1α and IL-1β are produced in the lungs during *B. pertussis* infections, the roles of these specific molecules in the clearance of the bacteria has not been determined [10], [27], [40]. To define the roles of each subtype of IL-1, we inoculated *B. pertussis* by the intranasal route into wild-type mice and mice lacking IL-1α, IL-1β, or IL-1R. At days 3, 7, 14, and 28 post-inoculation, bacterial CFUs were determined from excised lungs. Early during the infection, no significant differences in bacterial load were observed between wild-type and the knockout mice (Figure 1). However, by day 14 post-inoculation, the bacterial burden recovered from IL-1α, IL-1β, and IL-1R deficient mice was significantly higher than that of wild-type mice (3.9-fold, 8.7-fold, and 4.0-fold higher than wild-type, respectively). On day 28 post-inoculation, the difference between wild-type and IL-1α deficient mice was no longer apparent; however, both the IL-1β (>220-fold higher than wild-type) and IL-1R deficient mice (>280-fold higher than wild-type) remained very significantly colonized (p<0.01) by *B. pertussis* (Figure 1). These data suggest that IL-1R-mediated signaling requires IL-1β but not IL-1α to efficiently clear *B. pertussis* infection.

### Increased inflammation in IL-1 deficient mice

**Figure 2:**
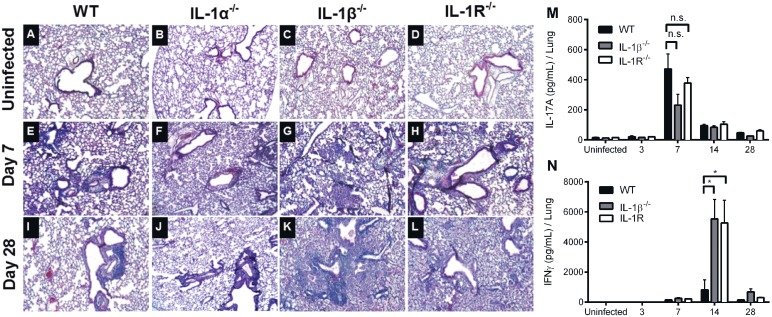
IL-1 signaling limits lung inflammation during *B. pertussis* infection. Groups of four mice were either (A–D) uninfected or inoculated with *B. pertussis* and euthanized at (E–H) 7 days or (I–L) 28 days post-inoculation. Automated H&E staining was performed on 5 µm paraffin-embedded sections. Images were taken and are representative of 10–12 fields per group. Lung homogenate cytokine measurements (M–N) were measured by ELISA from uninfected mice or on days 3, 7, 14, or 28 post-inoculation (n = 4–8). ELISA significance was determined by one-way ANOVA with Dunnet’s post-hoc test. *p<0.05 represents a significant difference from WT mice.

Given the critical role that IL-1 plays in initiating inflammation during infection we hypothesized that IL-1 deficient mice would have significantly higher bacterial-mediated tissue pathology in the lungs. Hematoxylin and eosin (H&E) stained sections of lungs of wild-type, IL-1α deficient, IL-1β deficient, and IL-1R deficient mice were analyzed in a blinded fashion (Figure 2A–L). On day seven post-inoculation, all examined mice showed pronounced bronchopneumonia (Figure 2E–H). By day 28, wild-type and IL-1α deficient lungs showed reduced alveolar exudate and fewer inflamed bronchioles suggesting resolution of inflammation consistent with the lower bacterial numbers in these mice. However, the majority of bronchioles of both IL-1β and IL-1R deficient mice remained inflamed with diffuse bronchopneumonia (Figure 2I–L, S1). Particularly, the intra-alveolar inflammation in the lungs of IL-1β deficient and IL-1R deficient mice was markedly higher than that of wild-type mice. This suggests that IL-1R mediated signaling is not required for initiation and establishment of inflammation in the lung but may be required for subsequent resolution of inflammation.

A recent report examining the contribution of Th1 and Th17 cells in adaptive immunity to *B. pertussis* showed involvement of both T helper subsets in protective immunity following primary infection and whole-cell vaccination [41]. To determine whether these responses were dependent on IL-1R signaling, as was suggested in previous publications, we measured lung cytokine responses during infection [10], [42]. Our data show that primary infection induces significantly higher amounts of IFNγ in IL-1β and IL-1R deficient mice than in wild-type mice on day 14 post-inoculation. We did not detect any significant difference in the amount of IL-17A produced by wild-type, IL-1β deficient and IL-1R deficient mice (Figure 2M–N). It should be noted, however, that a trend (IL-1β^−/−^ p = 0.08, IL-1R^−/−^ p = 0.4) toward lower IL-17A was observed in the IL-1 deficient mice (Figure 2M). These findings demonstrate that primary infection is capable of producing potent pro-inflammatory responses independent of IL-1R signaling but that these responses are not sufficient for normal clearance of *B. pertussis.*

### IL-1 is not required for recruitment of immune cells to lung

**Figure 3:**
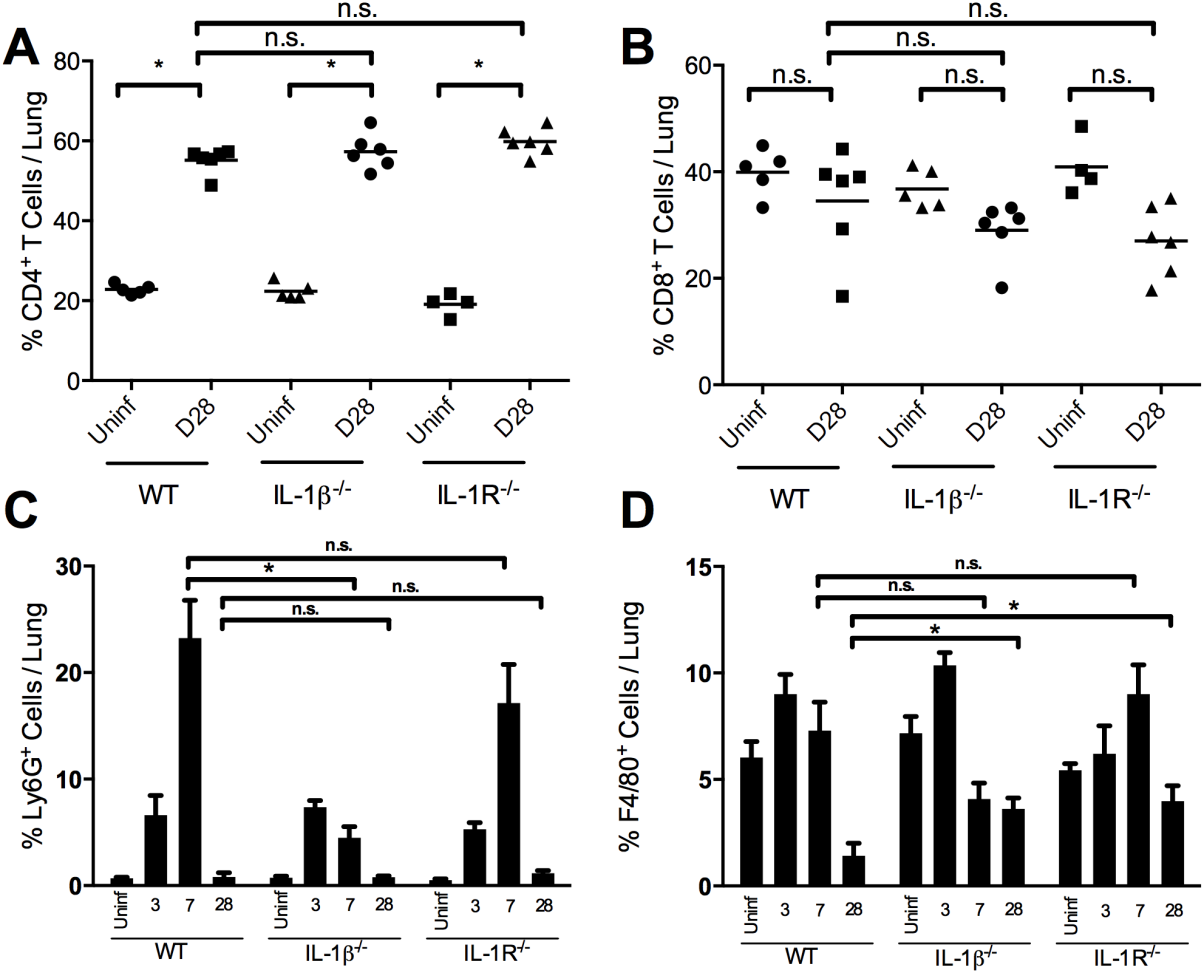
IL-1 is not required for recruitment of immune cells to the lung. Lungs were excised and made into a single cell suspension for flow cytometry at the indicated days post-inoculation. Cells were stained with anti-CD45 to identify hematopoietic cells and anti-CD11b, CD3, CD4, CD8, Ly6G, and F4/80 for further subsets. Gating for T cells subsets was performed on CD45^+^CD3^+^ cells with quadrant gating to identify either (A) CD4^+^ or (B) CD8^+^ single-positive T cells. Gating on CD45^+^CD11b^+^ cells, (C) Ly6G^+^ neutrophils or (D) F4/80^+^ macrophages were identified. Data was collected with a BD LSRII flow cytometer. Bars indicate the mean percentage of each group (n = 6) with (*) indicating p<0.05 using the Kruskal-Wallis test with Dunn’s post-hoc test for multiple comparisons.

During *B. pertussis* infection, initial inflammatory events lead to the recruitment of macrophage and dendritic cell populations followed by a peak of neutrophils approximately seven days post-inoculation, when bacterial numbers are also highest [37]. At this time point bacterial numbers begin to decline and macrophages and neutrophils present in the lungs begin to be replaced by CD4^+^ T cells as the adaptive immune response develops and spurs the production of IgA and IgG which are involved in clearance of *B. pertussis* [37]. To investigate whether IL-1 is required for the differences in cells present during infection, we inoculated mice with *B. pertussis* and collected the total lung homogenate and analyzed T cell subsets, neutrophils, and macrophages by flow cytometry. CD8^+^ T cells comprised 30–40% of total immune cells and were not increased by *B. pertussis* infection. In contrast, CD4^+^ T cells increased in proportion from approximately 20% to approximately 60% of total immune cells in wild type, IL-1β-deficient and IL-1R-deficient mice (Figure 3A, B). Except for day 7 post-inoculation in the IL-1β deficient mice, no significant defects were observed between mouse strains in the proportion of neutrophils during infection (Figure 3C). By day 28, the proportions of neutrophils in infected wild-type, IL-1β deficient, and IL-1R deficient mice were similar to those of uninfected mice (Figure 3C). Despite harboring more bacteria in the lungs throughout the infection, similar proportions of macrophages are present in the lungs of IL-1β and IL-1R deficient mice until 28 days post-inoculation when a higher proportion of CD11b^+^F4/80^+^ macrophages were recovered from the lungs of IL-1β and IL-1R deficient mice (Figure 3D) positively correlated with CFUs (r^2^ = 0.445, 0.842 respectively). A likely explanation for this persistence of macrophages is persistent infection in the IL-1-deficient mice. This also suggests that IL-1 signaling may contribute to the bacterial killing ability of macrophages during *B. pertussis* infection.

### Caspase-1 independent IL-1β mediates clearance of *B. pertussis*

**Figure 4:**
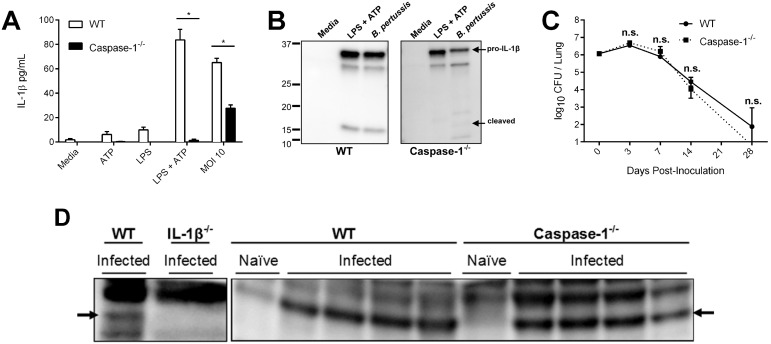
IL-1β production is caspase-1 dependent in vitro but independent in vivo. To determine if *in vitro* IL-1β processing is dependent upon caspase-1, bone marrow-derived macrophages were (A) primed 3 h prior to inoculation and were washed and treated with fresh media, ATP (5 mM), or live *B. pertussis* (MOI 10) or (B) seeded at 10^6^ cells/well and primed with LPS or washed and treated with media, ATP (5 mM), or live *B. pertussis* (MOI 100) for 6 hours in FBS-free DMEM, and supernatant was collected. The requirement for caspase-1 during infection and the ability to produce IL-1β in response to live *B. pertussis* was assessed by (C) inoculating groups of four mice per time point with *B. pertussis* (5×10^5^ CFU) and lungs were dissected for determining bacterial CFUs ± standard error. (D) To identify whether cleaved IL-1β was produced in caspase-1 deficient mice, lung homogenates from day 3 post-inoculation were run on 12% gels and probed with anti-IL-1β. Data is representative of at least two independent experiments.

Previous data has shown that the NLRP3/caspase-1-containing inflammasome is activated by the *B. pertussis* adenylate cyclase toxin through formation of a pore in the host cell membrane and efflux of K^+^ ions [10]. Given that IL-1β mediates the efficient clearance of *B. pertussis* from the lungs, we also expected the inflammasome to be required for control of the primary infection [10]. To examine the direct role for caspase-1-dependent IL-1β secretion in response to live *B. pertussis,* we generated BMDMs from wild-type and caspase-1 deficient mice and exposed each to *B. pertussis* at an MOI of 10 for 24 hours. Consistent with previous publications, *in vitro* LPS/TLR4 stimulation alone was insufficient for inflammasome-dependent secretion of IL-1β but required an additional signal such as ATP (Figure 4A, B), which causes an efflux of intracellular K^+^ and activation of the NLRP3/caspase-1 inflammasome [43], [44]. Infection of caspase-1 deficient BMDMs with live *B. pertussis* induced release of IL-1β into the culture supernatant, although at significantly lower levels than wild-type cells (Figure 4A). These supernatants were precipitated from wild-type and caspase-1 deficient BMDMs infected with *B. pertussis*, blotted, and probed with anti-IL-1β antibody to determine whether active IL-1β was secreted. Only precursor IL-1β was observed in caspase-1 deficient macrophages suggesting that caspase-1 is still required for cleavage and secretion of mature IL-1β in *in vitro* culture conditions (Figure 4B).

To determine whether inflammasome activation is required for the generation of mature IL-1β in the lungs during primary infection, wild-type and caspase-1 deficient were intranasally inoculated with *B. pertussis* (5×10^5^ CFU) and lungs were homogenized and serially diluted on BG-blood agar. Surprisingly, bacterial numbers were similar in wild-type and caspase-1 deficient mice, indicating that caspase-1 is not required for clearance of *B. pertussis* from the lungs (Figure 4C). Since caspase-1 is also required for the processing of IL-18 [7], these data suggest that IL-18 is also not required for clearance of a primary *B. pertussis* infection. This was confirmed by inoculation of IL-18 deficient mice, which controlled and cleared *B. pertussis* as efficiently as wild-type mice (data not shown). To determine whether mature IL-1β was generated at the site of infection, murine lung homogenates harvested on day 7, at the peak of infection and IL-1 expression, were probed for mature and pro-IL-1β by immunoblotting. Interestingly, the mature form of IL-1β was observed in wild-type and caspase-1 deficient mice (Figure 4D). Together these data indicate that during *B. pertussis* infections of the lung, caspase-1-dependent activation of IL-1β is not necessary and suggests the involvement of other enzymes.

### Adaptive immune responses are IL-1 dependent but caspase-1 independent

**Figure 5:**
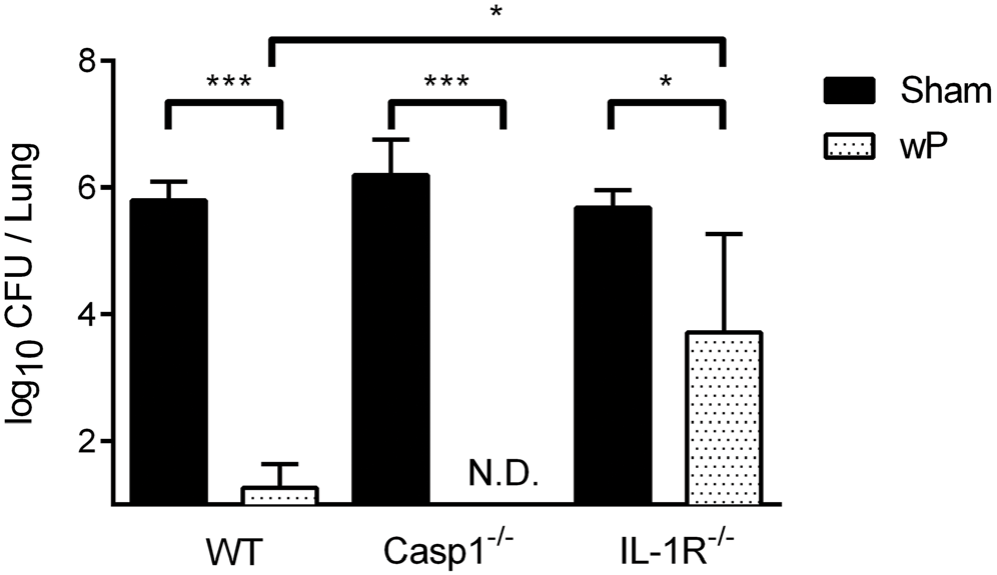
Adaptive immune responses are IL-1 dependent but Caspase-1 independent. Groups of four mice were either sham vaccinated with PBS or vaccinated with heat-killed *B. pertussis* (wP) by administering 200 µL *i.p.* at 28 and 14 days prior to challenge inoculation with 5×10^5^ CFU. Lungs were excised at seven days post-challenge and to determine bacterial CFUs. Bar graphs represent the mean CFU per lung ± standard deviation. Significance was determined by one-way ANOVA and Tukey’s multiple comparison post-hoc test with *p<0.05, ***p<0.001. Data is representative of two independent experiments.

Previously we have shown IL-1R-mediated signaling to be important for the rapid clearance of *B. pertussis* from the lungs of mice given an acellular *B. pertussis* vaccine [27]. A recent study also showed that the current acellular vaccine acts through promoting a NLRP3-independent IL-1 response that generates robust Th1 and Th17 responses, a phenomenon also observed during primary infection [41]. However, the mechanism behind whole-cell vaccination is not known. To determine whether protective immunity is dependent on any alternate caspase-1-dependent inflammasomes, we vaccinated animals by administering two doses of heat-killed *B. pertussis* antigens intraperitoneally 28 and 14 days prior to a challenge inoculation. All unvaccinated animals had approximately 10^6^ CFU of *B. pertussis* three days after challenge. Vaccination allowed both wild type and caspase-1 deficient mice to completely clear *B. pertussis* infection within seven days, indicating that caspase-1 is not required for the generation of protective immune responses to a whole cell vaccine. Vaccinated IL-1R deficient mice failed to clear *B. pertussis* and still had approximately 10,000 CFU in their lungs, indicating that IL-1R signaling is required for the efficient generation and/or function of protective immunity (Figure 5). Neither IL-1β nor IL-1R was required for the generation of antibodies specific for *B. pertussis* (data not shown) suggesting a role in inflammation and/or cellular responses. These findings suggest that the sterilizing memory response requires IL-1R-mediated signaling but not caspase-1.

## Discussion

Experimental infections of mice lacking inflammasome components have shown that the inflammasome has an essential role in generating protective immune responses against multiple pathogens ranging from viruses to bacteria to fungi [45], [46]. Depending on the pathogen, one or more specific NLRs are recruited to the inflammasome complex which leads to activation of caspase-1 and secretion of mature IL-1β and IL-18 [47]. Previous work has suggested that infection with *B. pertussis* leads to activation of the NLRP3/caspase-1 inflammasome and this activation depends on the pore-forming activity of the adenylate cyclase toxin [10]. These observations were demonstrated *in vitro* with bone marrow-derived dendritic cells (BMDCs) and in this study in bone marrow-derived macrophages (BMDMs) [10]. These results are consistent with numerous studies indicating that inflammasome activation is required for mature IL-1β in *in vitro* models. In contrast, infection models utilizing *Mycobacterium tuberculosis* and *Pseudomonas aeruginosa* have shown that while IL-1R signaling is required to control infection, caspase-1 deficient mice are capable of generating mature IL-1β and clearing the infections, stressing the importance of using animal models for host-pathogen interaction studies [48]–[50]. Similarly, our studies have shown that mature IL-1β could be detected in caspase-1 deficient mice and these mice are capable of clearing *B. pertussis* infection. Interestingly, infections in caspase-1/11 deficient mice showed no defect in bacterial clearance, suggesting that the protective IL-1β response in the lung can arise independently from these inflammasomes.

Host IL-1 signaling generally leads to robust Th1 and Th17 responses which have an important role in activating phagocytes and multiple other inflammatory pathways. In this study, we examined the upstream processing and signaling that generates this protective response to *B. pertussis*, observing no role for IL-1α but a significant role for IL-1β and its downstream IL-1R-mediated signaling. We also determined that IL-18, another cytokine that is classically caspase-1 dependent, is not required for clearance of primary *B. pertussis* infections (data not shown). Further, while previous studies showed a defect in bacterial clearance in IL-17A and IL-17R knockout mice, we observed no significant defect in the production of this cytokine in the lungs of IL-1β/IL-1R deficient mice during a primary infection, suggesting these cytokines are not sufficient for *B. pertussis* clearance in the absence of IL-1R signaling [10], [41], [42]. These findings are significant because recent work has shown that protective Th17 responses require IL-1R signaling when alum is used as an acellular vaccine adjuvant but do not require IL-1R during a natural infection [41].

The effect of IL-1 during infection has been attributed to its role in activating the recruitment of immune cells to local sites of infection through the upregulation of chemokines and cytokines. However, histological examination shows that IL-1β/IL-1R deficient mice are able to recruit inflammatory cells to the same extent as that of wild type mice. In addition, the persistence of diffuse inflammation in IL-1 deficient mouse lungs is correlated with the bacterial burden. This finding is similar to previous work using another IL-1R deficient mouse strain (B6; 129S1-*Il1r1^tm1R^* ^o*ml*^/J) in which *B. pertussis* caused death but also highlights that there may be significant mutation and strain-specific effects of IL-1 signaling [27]. Seven days post-inoculation we also observed a reduced percentage of neutrophils in IL-1β deficient mice, but this was not observed in IL-1R deficient mice. This may suggest the early defect in neutrophil recruitment in IL-1β deficient mice may impact bacterial clearance during the latter part of the time-course and resolution of inflammation. However, we observed a similar amount of neutrophil recruitment in IL-1R deficient mice compared to wild-type, suggesting neutrophil recruitment may not impact bacterial clearance in the latter portion of the infection. We are currently investigating the early neutrophil defect in the infection process. These data would suggest that while IL-1 signaling is not required for recruitment of cells, the activation of robust bacterial killing mechanisms during primary infections does require IL-1β. This cytokine most likely plays a role in overcoming the effects of pertussis toxin and adenylate cyclase toxin, which have been shown to inhibit neutrophil and macrophage antimicrobial defenses [27], [37], [51], [52].

During the immune response to pathogens, inflammatory sites are often dominated by immune cells with diverse sets of secreted enzymes that have been shown to be involved in cleaving proteins or the extracellular matrix [53], [54]. Because caspase-1 is not required for the maturation of IL-1β in the lungs of infected mice, alternative host-associated factors are likely responsible. Known enzymes include the serine proteinase 3 (PR3), neutrophil elastase (NE), cathepsin G, matrix metalloproteases (MMPs), stromelysin-1, gelatinases A and B, and caspase-8, which have all been shown to be capable of cleaving pro-IL-1β into a biologically active form *in vitro* or in the mouse model [20]. Indeed, during arthritis-induced sterile inflammation, caspase-1-independent processing of IL-1β leads to disease-associated inflammation as a result of neutrophil recruitment and proteinase 3, while *P. aeruginosa* infections of the cornea require neutrophil elastase [49], [55]. Therefore, we speculate that neutrophil or macrophage-derived enzymes present during the peak of *B. pertussis* infections, when IL-1β expression and immune cell recruitment also peak, could lead to the processing of IL-1β in the lung, but the specific mechanism remains to be examined.

Another important outcome of infection is the generation of a protective adaptive immune response. Natural immunity following infection has been shown to be more protective than the current acellular vaccines, so understanding which molecular signals generate this enhanced protection is important for improving future vaccines [28], [29]. Here we have demonstrated that the caspase-1 inflammasome is not required to generate a protective immune response following vaccination using heat-killed *B. pertussis* as a vaccine though IL-1 signaling is required [27]. This is consistent with a recent publication showing that the acellular vaccine protects NLRP3 deficient mice from challenge infections [41]. The latter study does not rule out the requirement for caspase-1 in the generation of protective immunity as other NLRs may be involved. However, no other NLRs have been shown to be involved in recognizing *B. pertussis* thus far. Our work, on the other hand, provides definitive proof that caspase-1 and thus inflammasome-dependent IL-1 is not required for generating immunity from a heat-killed vaccine. Taken together, these data suggest that protective *B. pertussis* vaccines could function through activation of the IL-1R in an inflammasome-independent manner during the recall response that acts on antigen-specific Th1 and Th17 cells, similar to the response seen during a primary infection.

Overall, our work shows that the IL-1β/IL-1R axis is required, independent from inflammasome activity, to resolve *B. pertussis* infections in the mouse model. Interestingly, we have also observed that a closely related bacterium *B. bronchiseptica*, which shares over 90% of its genome with *B. pertussis* and is consistently more virulent in mouse models, is cleared normally in IL-1R or caspase-1 deficient mice (unpublished data). IL-1R signaling was not required for the control of *B. parapertussis* infection, suggesting some unique aspect of *B. pertussis* results in a particular need for the IL-1R signal [27]. We propose that inflammasome-independent IL-1 signaling, in addition to robust Th1, Th17, and antibody responses, synergize to overcome the effects of *B. pertussis* pertussis toxin and adenylate cyclase toxin that impair the phagocytic capacity of macrophages in the lung. These findings highlight the need to further understand how bacterial virulence factors interact with and can be overcome by the host immune response during infections.

## Supporting Information

Figure S1 **Lung pathology scoring of** ***B. pertussis*** **infection.** Groups of mice (n = 3–6) were inoculated and lungs were collected for histology. (A) Peribronchiolar (degree and % lung affected combined), (B) perivascular, and (C) intra-alveolar inflammation were scored (0–4) and averaged from two blinded, independent scorings. Bars indicate mean score of each group.
(TIF)
