# Supplementary figures and images for: Caspase-1-Independent Interleukin-1β Is Required for Clearance of Bordetella pertussis Infections and Whole-Cell Vaccine-Mediated Immunity
Source: PLoS One. 2014 Sep 8;9(9):e107188. doi: 10.1371/journal.pone.0107188 (PMC4157866; doi:10.1371/journal.pone.0107188)

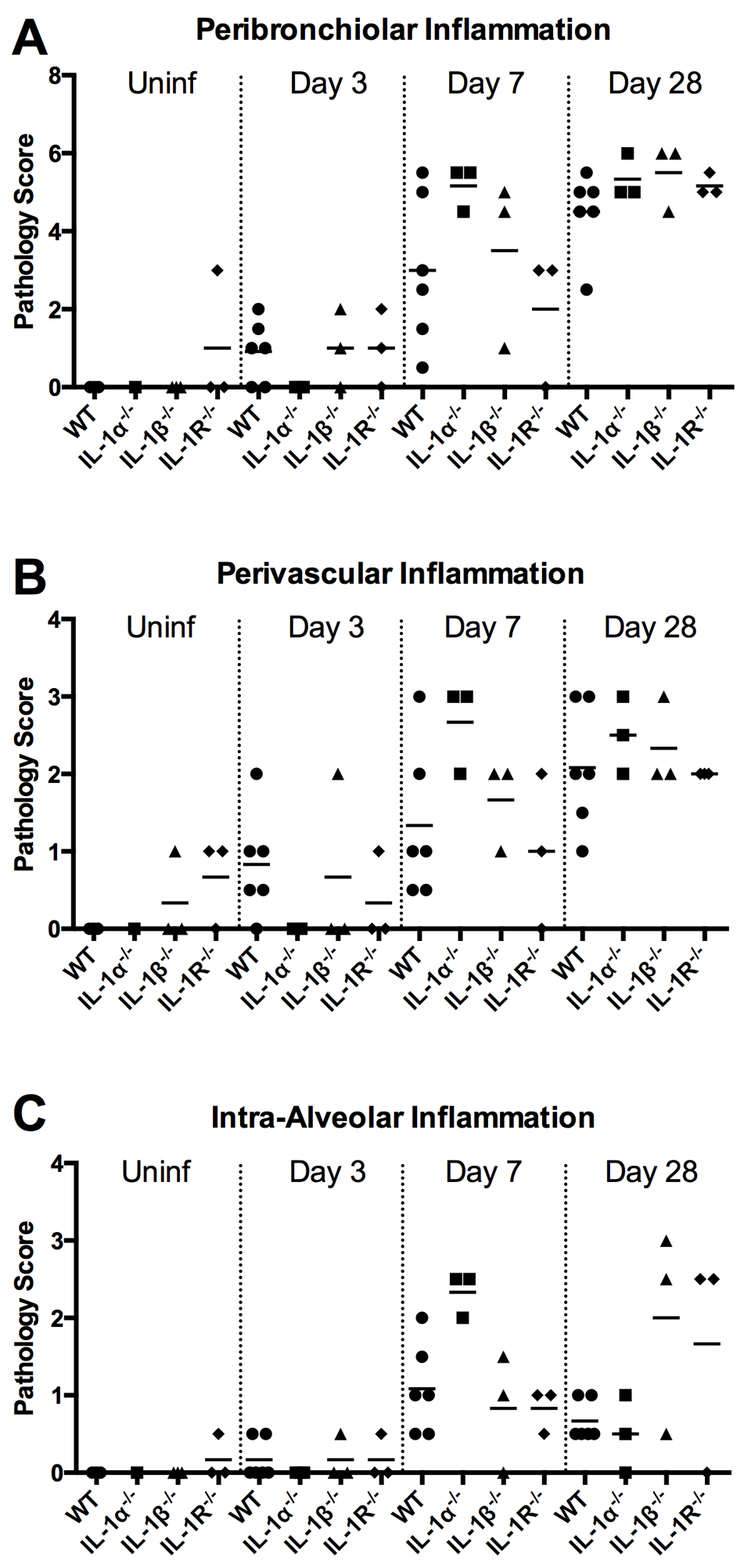

Supplement: Figure S1 — Lung pathology scoring of B. pertussis infection. Groups of mice (n = 3–6) were inoculated and lungs were collected for histology. (A) Peribronchiolar (degree and % lung affected combined), (B) perivascular, and (C) intra-alveolar inflammation were scored (0–4) and averaged from two blinded, independent scorings. Bars indicate mean score of each group. (TIF) [file pone.0107188.s001.tif]
